# Presleep Ruminating on Intrusive Thoughts Increased the Possibility of Dreaming of Threatening Events

**DOI:** 10.3389/fpsyg.2022.809131

**Published:** 2022-01-26

**Authors:** Xiaoling Feng, Jiaxi Wang

**Affiliations:** ^1^School of Psychology, South China Normal University, Guangzhou, China; ^2^Institute of Analytical Psychology, City University of Macao, Macao, China

**Keywords:** continuity hypothesis, content analysis, dreaming, rumination, threat simulation theory

## Abstract

This study investigated whether ruminating on an intrusive thought before sleeping led to an increased likelihood of dreaming of threatening events. One hundred and forty-six participants were randomly assigned to a rumination condition (a rumination on an intrusive thought for 5 min before sleeping; *N* = 73) and a control condition (think about anything for 5 min before sleeping; *N* = 73). Participants completed a dream diary upon waking. The result showed that presleep ruminating on an intrusive thought increased the frequency of both threatening dreams and negative emotions in dreams. In addition, dreams with threatening events were more emotional and negative than dreams without threatening events. These results may support the threat simulation theory of dreaming. In addition, these results may give some insight into a mathematical model for the continuity hypothesis of dreaming.

## Introduction

Dreaming is the subjective experience during sleep (e.g., see Schredl, 2018). It is proposed to be a simulation of the waking world, of waking reality, or of waking consciousness. Thus, in general, dreaming can be defined as a simulated world (e.g., Revonsuo, 2000; Revonsuo et al., 2015; Domhoff, 2019).

Hall in his works argues that there is a continuity hypothesis (CH; e.g., Hall and Nordby, 1972), which suggests that “*dreams are continuous with waking life; the world of dreaming and the world of waking are one. The dream world is neither discontinuous nor inverse in its relationship to the conscious world. We remain the same person, the same personality with the same characteristics, and the same basic beliefs and convictions whether awake or asleep. The wishes and fears that determine our actions and thoughts in everyday life also determine what we will dream about. How can we reconcile the continuity hypothesis with the obvious fact that a person will do something in his dreams that he would not or could not do in a waking state? He will, for example, torture someone to death, have sex with his young daughter, betray his best friend, or fly through the air. The answer to this dilemma is to be found in the distinction between overt behavior (“acting out”) and covert behavior (thoughts, feelings, and fantasies). The continuity may be between dreams and covert behavior or it may be between dreams and overt behavior. A person who has many sex or aggression dreams may either have many fantasies of sex or aggression when he is awake, or he may have many actual sexual or aggressive experiences. In either case he is preoccupied with sex or aggression, awake or asleep. Although when asleep these preoccupations have fewer limitations, allowing the dreamer to experience tremendous diversity in his sexual and aggressive fantasies*” (Hall and Nordby, 1972, p. 104). Domhoff (1996, 2001, 2017) emphasizes the cognitive aspect of the CH in his works, which argues that dreams express the same conceptions and personal concerns that animates waking thought. This version of the CH is a kind of simulation proposal that focuses on the continuous nature of cognitive aspects between waking and dreaming (see Domhoff, 2017). By contrast, the original version of the CH indicates not only the cognitive aspect of continuity between waking and dreaming but also the influence of daily-life activities on dreams (see above).

Compared with Domhoff (1996, 2001, 2017), Schredl (2003) in his works cites the CH in a closer way to Hall’s idea (e.g., Hall and Nordby (1972)), according to which the CH means that dreaming is in continuity with waking life. Further, Schredl (2012) concludes that many different aspects of waking life can show up in dreams, such as activities, concerns, emotions, and thoughts. Citation of Schredl for the CH has been termed as the incorporation continuity hypothesis (ICH; e.g., Domhoff, 2017; Tuominen et al., 2019). In the following, we still adopted the term ICH to represent formulation of Schredl (2003, 2012) for the CH Hall and Nordby (1972).

The ICH has been criticized that any presence of continuity between waking and dreaming can be seen as supporting the ICH (e.g., Tuominen et al., 2019). In addition, the ICH cannot explain why some dreams are not continuous with waking-life experiences (e.g., see Hobson and Schredl, 2011; Horton, 2017). In our view, the important value of the ICH is that it suggests that waking-life experiences can influence subsequent dreams. However, the limitation of the ICH is that its definition for the effect of waking-life experiences on dreams is “vague and broad” (Schredl, 2003, p. 1). The cognitive version of the CH (e.g., Domhoff, 2017, 2019), on the other hand, has an essential value because it points out that dreams are the simulation of the waking world, which means there is a continuous conception and concerns between waking and dreaming. This kind of proposal narrows down the range of the CH to the cognitive aspect. However, the cognitive version of the CH does not describe the phenomenon that waking-life experiences can affect subsequent dreams (e.g., see Domhoff, 2020).

The threat simulation theory of dreaming (TST; Revonsuo, 2000) is a theory that describes threatening events in dreams. According to the TST, during dreaming, the experience of real threatening events triggers the activation of the threat simulation system (TSS) and the latter will have recourse to elements drawn from long-term autobiographical memory (LTAM; e.g., see Valli and Revonsuo, 2009). The situations encounters in dreams help us learn how to recognize and respond to threatening circumstances. The better we get at applying this knowledge to real threats in our waking life, the more likely we are to survive and reproduce. So dreaming has an evolutionarily adaptive function. In explaining threatening dreams, the TST contains the value of both the ICH and the cognitive version of the CH. On the one hand, similar to the cognitive version of the CH, the TST is a kind of simulation theory, suggesting that dreams are simulations of the waking world. On the other hand, similar to the ICH, the TST suggests that waking-life experiences can affect subsequent dreams.

Concerning the topic of the influence of threatening waking-life experiences on dreams, the TST states that the experience of real threats triggers the activation of the TSS that activates long-term threatening memories during dreaming (Valli and Revonsuo, 2009). Some studies supported the TST, by showing that the presence of threatening waking-life experiences was correlated with the frequency of threatening dreams (Bradshaw et al., 2016; Lafrenière et al., 2018). However, these studies were correlational. Here we tried to explore this topic experimentally.

According to Schredl (2003), some presleep experimental manipulation for waking-life experiences can affect subsequent dreams. For example, presleep watching an erotic film enhanced the possibility of dreaming of content related to erotic content (Cartwright et al., 1969). Presleep emotional suggestion affected subsequent dream emotions (De Koninck and Brunette, 1991). Presleep focusing on personal issues enhanced the possibility of dreaming of concern-related material (e.g., Saredi et al., 1997; Nikles et al., 1998). In addition, the influence of presleep consideration for newly acquired stimuli on increased the possibility of dreaming of the stimuli (Cipolli et al., 2004).

Recently, some studies found that suppressing an intrusive thought before falling asleep enhanced the possibility of dreaming of the thought (e.g., Taylor and Bryant, 2007; Bryant et al., 2011). This kind of finding implied that presleep manipulation for intrusive thoughts could affect subsequent dreams. The intrusive thought referred to negative and distressing thoughts that popped into one’s mind involuntarily and spontaneously. Thus the intrusive thought may be a kind of threatening event. According to the TST, encountering threatening events during waking may lead to dreams with threatening events. There is evidence suggesting that ruminating on intrusive thoughts may trigger more intrusive memories (Smets et al., 2012). So if a person ruminates on one’s intrusive thought before falling asleep, the person may have more chance to dream of threatening events caused by the intrusive thought. Here we explored this topic, by hypothesizing that presleep ruminating on intrusive thoughts increased the possibility of dreaming of threatening events.

## Materials and Methods

### Participants

One hundred and sixty participants were selected to participate in this study. All of them were college students in Guangzhou province, China. Participants were accepted for the study if they were self-reported to be sufficient with the following criteria: recall at least two to three dreams per week; sleep at least 6 h per night; take no more than 30 min to fall asleep; have no neurological or psychiatric history; non-smokers. Also, participants were asked not to take recreational drugs and alcohol during the experiment period. The participants were equally divided into two groups at the time of signing up in an alternating fashion. Participants were unknown that they had been assigned to a group. However, 11 of them were not able to recall their night dreams (five from the rumination group; six from the control group), and three of them did not finish their night task in the evening, so these participants were not included in the research, reducing the number of participants to 146: the rumination group [*N* = 73; 15 males, 58 females; mean (SD, range) age 21.36 (2.21, 18–29) years], and the control group [*N* = 73; 13 males, 60 females; mean (SD, range) age 21.09 (2.18, 18–28) years]. All subjects gave written informed consent before the start of the study, and the local Research Ethics Committee approved the study.

### Night Task

In the “Night Task” participants were instructed to identify and describe in detail their most negative and distressing intrusive thought. The definition of an intrusive thought follows Taylor and Bryant (2007): “*a thought or image that you do not intend to think about, but pops into your head every now and then without you wanting it to. It may be a thought or image about a particular person, object, place, past event, imagined future event or even about yourself. It must be a thought or image that you have had before on more than one occasion. For the purpose of this study, it should be a thought or image that you do not like and one that you do not enjoy having intrude into your mind.*” Participants also rated the level of distress caused by the intrusive thought on a five-point Likert (1 = not at all distress, 5 = extremely distress) and indicated how many days in the past week they had their nominated target thoughts.

In the rumination condition, participants were instructed to engage in a 5-min stream of consciousness writing task. They were instructed to write down all their thoughts but were explicitly instructed to think about the distressing thought. The specific instruction was adapted from Bryant et al. (2011): You are to write down all the thoughts you are having as they come into your mind. You can think of anything you like: who you spoke to today, whatever you are doing tomorrow, your favorite holiday. But you should try your best to think about the intrusive thought you identified above, even only for a second. Do whatever it takes to keep that thought in your mind. Think of the thought. I cannot stress enough how important it is that you allow the thought to enter your mind for the 5 min you are completing this stream of consciousness writing task. Each time the intrusive thought does pop to mind, please make a checkmark on the page just as you were shown to do in the practice exercise.

In the control condition, participants followed a similar instruction to the instruction in the rumination condition, except participants were not instructed to think about the distressing thought. The specific instruction follows Bryant et al. (2011): “*You are to write down all the thoughts you are having as they come into your mind. You can think of anything you like: who you spoke to today, whatever you are doing tomorrow, your favourite holiday, even the intrusive thought you identified above. Do not try to control your thoughts at any stage, simply let them come and go as they please, even the intrusive thought you identified before. Do whatever it takes to think about any thoughts. Think of anything you want, anything at all. I cannot stress enough how important it is that you allow any thoughts to enter your mind for the 5 min you are completing this stream of consciousness writing task. Each time the intrusive thought does pop to mind, please make a check mark on the page just as you were shown to do in the practice exercise.*” The distressing memory was mentioned the same number of times in both instructional sets to equalize possible priming of the memory.

### Morning Task

In this task, participants were instructed to record their dreams from the previous night: Describe everything in your dreams, with as much detail as possible, similar to a narrative story: what happened, in what time frame, with whom, and so forth. Describe the cognitions, emotions, and behaviors you experienced in your dream, as well as the cognitions, emotions, and behaviors of all other parties included in your dream (if evident to you). If it was a lucid dream, state so. In addition, participants rated the level of the pleasantness of the dream (1 = very unpleasant, 5 = very pleasant), the level of the emotional intensity of the dream (1 = not at all intense, 5 = extremely intense).

### Dream Content Analysis

The collected dreams were randomized and scored by two independent raters (both psychological postgraduates) blind to the subject variables. The raters worked independently, using operationalized definitions to classify if there was any threatening event in dreams. The method to rate threatening events follows Valli et al. (2005): 1. Escape and pursuits; 2. Accidents and misfortunes; 3. Failures; 4. Catastrophes; 5. Disease and illness; 6. Aggression; 7. Non-physical aggression; and 8. Direct physical aggression. A score of 0 for no presence or 1 for the presence of threatening events in dreams was used.

### Procedure

As Bryant et al. (2011) showed a small-to-medium effect size for the dream rebound effect, we adopted a similar effect size to calculate the sample size. Based on the small-to-medium effect size (phi = 0.25), *a priori* power analysis for chi-square analysis was conducted with G*Power 3 to predict the sample size required. It was estimated that approximately 126 participants would be required with an alpha level of 0.05 and power of 0.80.

Participants first received an eligibility questionnaire (see Section “Participants”) and an information sheet. If they met the criteria and agreed to take part, they received the instructions and links they needed to take part *via* email. Participants finished the experiment *via* an online questionnaire web, using their own devices, such as a telephone or a computer. They were randomly assigned to one of two conditions: a rumination condition or a control condition. Participants were given two online instructions: one labeled “Night Task” and the other labeled “Morning Task.” They were instructed to open and complete the “Night Task” contents directly before going to sleep on a night of their choice in the coming week (detail see Section “Night Task”). Participants were instructed to complete the “Morning Task” immediately after waking (detail see Section “Morning Task”). Dreams were subsequently coded by the two external judges who were blind to participants’ information. Cronbach’s consistency coefficient (*α*) among the two raters was 0.85. All inconsistencies between the two raters were discussed until reaching an agreement.

## Results

Participant characteristics, ratings of target thought, and ratings of dreams were reported in Table 1. At first, Wilcoxon rank-sum tests were used. Participants in the rumination condition (the rumination group) reported more intrusive thoughts during the stream of consciousness task than participants in the control condition (the control group; *z* = 4.39, *p* < 0.001), which suggested our manipulation was effective. The dream valence of the rumination group was more negative than the dream valence of the control group (*z* = 2.23, *p* = 0.026). The two groups did not differ on frequency of intrusive thought, the distress of intrusive thought, dream emotional intensity, dream length, and dream number (all *p* > 0.27).

**Table 1:** Mean (SD) participant characteristics, ratings of target thought, and ratings of dream content.

	Rumination condition^a^	Control condition^a^
Distress of intrusive thought	4.25 (0.68)	4.20 (0.71)
Frequency of intrusive thought	3.66 (1.92)	3.70 (1.93)
Number of presleep intrusions	5.86 (3.68)	3.41 (1.85)
Dream length	143.44 (141.54)	148.16 (139.27)
Number of dreams	1.21 (0.51)	1.16 (0.52)
Dream valence^b^	2.34 (0.85)	2.73 (0.88)
Dream emotional intensity^c^	3.34 (0.73)	3.38 (0.78)

a The number of participants in the rumination condition is 73, and the number of participants in the control condition is 73.

b 1 = very unpleasant, 5 = very pleasant.

c 1 = not at all intense, 5 = extremely intense.

For the rumination group, 72.6% (*N* = 53) dreams were threatening, whereas, for the control group, 46.6% (*N* = 34) dreams were threatening. A chi-square test showed that the rumination group reported more threatening events in dreams than the control group (*χ*^2^ = 10.27, *p* = 0.001, phi = 0.27, df = 1). So this result indicated that presleep ruminating on intrusive thoughts increased the possibility of dreaming of threatening events.

In addition, for each kind of condition, we compared potential differences between participants who reported threatening events in dreams (the threatening dream group) and participants who did not report threatening events in dreams (the non-threatening dream group). Participant characteristics, ratings of target thought, and ratings of dreams were reported in Table 2. For the rumination condition, the threatening dream group reported more emotional (*z* = 3.22, *p* = 0.001), more negative (*z* = 2.89, *p* = 0.004), and longer (*z* = 2.65, *p* = 0.008) dreams than the non-threatening dream group. The two groups did not report any difference for other kinds of variables: the number of presleep intrusions, the frequency of intrusive thought, the distressing of the intrusive thought, and dream numbers (all *p* > 0.32). For the control condition, the threatening dream group reported more emotional (z = 2.67, *p* = 0.008), more negative (*z* = 2.82, *p* = 0.005), and longer (*z* = 3.76, *p* = <0.001) dreams than the non-threatening dream group. The two kinds of groups did not report any difference for other kinds of variables: the number of presleep intrusions, the frequency of intrusive thought, the distressing of the intrusive thought, and dream numbers (all *p* > 0.16). In short, the above results showed that threatening dreams were more emotional, more negative, and longer than non-threatening dreams.

**Table 2:** Mean (SD) participant characteristics, ratings of target thought, and ratings of dream content.

	Rumination condition		Control condition	
	Threatening dream group^a^	Non-threatening dream group^a^	Threatening dream group^a^	Non-threatening dream group^a^
Distress of intrusive thought	4.26 (0.68)	4.20 (0.70)	4.15 (0.74)	4.26 (0.68)
Frequency of intrusive thought	3.58 (1.84)	3.85 (2.13)	3.82 (2.07)	3.59 (1.82)
Number of presleep intrusions	5.75 (3.50)	6.15 (4.19)	3.59 (1.58)	3.26 (2.07)
Dream length	152.81 (120.93)	79.40 (43.14)	209.71 (167.86)	97.80 (79.80)
Number of dreams	1.25 (0.52)	1.15 (0.49)	1.19 (0.53)	1.09 (0.49)
Dream valence^b^	2.17 (0.81)	2.85 (0.90)	2.38 (0.84)	3.03 (0.95)
Dream emotional intensity^c^	3.51 (0.70)	2.90 (0.64)	3.65 (0.73)	3.15 (0.74)

a For the rumination condition, the numbers of participants in the threatening dream group and the non-threatening dream group are 53 and 20 separately. For the control condition, the numbers are 34 and 39 separately.

b 1 = very unpleasant, 5 = very pleasant.

c 1 = not at all intense, 5 = extremely intense.

## Discussion

The dream valence of the rumination group was more negative than the dream valence of the control group. This result indicated that presleep ruminating on intrusive thoughts affected the possibility of dreaming of negative emotions. Smets et al. (2012) suggested that ruminating about the content of intrusive thoughts could exacerbate negative emotions. In this study, after the stream of consciousness writing task participants in the rumination condition might have more negative emotions caused by their intrusive thoughts than participants in the control condition. As a result, the former group dreamed more of negative emotions in dreams. The result was in line with Carpenter (1988), which found that the induction of negative stimuli before sleep enhanced the possibility of dreaming of negative experiences. In addition, our result may also align with the evidence that presleep stressful experiences (De Koninck and Koulack, 1975; Koulack et al., 1985) enhanced the possibility of dreaming of negative experiences, because the ruminating process can cause stressful feelings.

We found that presleep rumination on intrusive thoughts increased the possibility of dreaming of threatening events. This result accorded with our hypothesis. In this study, intrusive thoughts were negative thoughts that participants did not like. These thoughts were distressful thoughts that people did not like. In addition, intrusive thoughts were thoughts that would appear in one’s mind unexpectedly. The two characteristics of intrusive thoughts were similar to threatening waking-life experiences. Lafrenière et al. (2018) found a correlation between the average severity of daytime threats and the presence of threats in dreams. In our study, the process of ruminating on intrusive thoughts might increase the severity of threats of the thoughts. Thus our results may be partly in line with Lafrenière et al. (2018). According to the TST (e.g., Revonsuo, 2000), the increased severity of threats activated the TSS, triggering threatening dreams. So presleep ruminating on intrusive thoughts increased the frequency of threatening dreams. Here our result may support the TST (e.g., Revonsuo, 2000).

In addition, we found that dreams with threatening events were more emotional and more negative than dreams without threatening events. It should be noted that the length of the former kind of dreams was longer than the length of the latter kind of dreams. In this study, participants rated their emotions in dreams immediately after they reported their dreams in the morning. So, participants might still remember clearly about their emotions in their dreams, and thus, the length of dreams might not affect the self-rating for dreams. Threats were negative toward people, so dreams with threatening events were more negative than dreams without threatening events. According to the TST (e.g., Revonsuo, 2000), threatening events during waking time trigger the TSS, activating salient long-term memories with threats. The threatening memories were memories with high severity of threats (high emotional intensity). So dreams with threatening events were more emotional than dreams without threatening events.

Moreover, according to the ICH (Schredl, 2003, 2012), dreaming is continuous with different aspects of waking life. Schredl in his work (Schredl and Hofmann, 2003) argues that the ICH in its present general form is not valid and should be specified more precisely. A way to address the problem is to study factors that can influence the incorporation rate of waking-life experiences (Schredl, 2003). The current study found that presleep ruminating on intrusive thoughts increased both negative emotions in dreams and the frequency of threatening events in dreams. These results were in line with Schredl and Hofmann (2003), which found that the amount of time spent with specific waking-life activities (e.g., driving a car) was associated with the possibility of dreaming of the waking-life activities. Thus our results may give some insight into mathematical model of Schredl (2003, 2012) for the ICH.

### Limitations and Future Directions

In the current study, intrusive thoughts were used as potential threatening waking events, but there were also other kinds of threatening events. So it was not clear if a ruminating on other kinds of threatening waking events could lead to a similar consequence. Nevertheless, the TST may predict that.

In addition, the TST was criticized because it could not explain dreams without threatening events (e.g., see Domhoff, 2019). Although we showed that presleep ruminating on intrusive thoughts enhanced the frequency of threatening events in dreams in this study, we did not explore if presleep focusing on neutral or positive waking-life experiences led to threatening dreams. Future studies could explore this topic.

## Conclusion

The current study found that presleep ruminating on intrusive thoughts enhanced the possibility of dreaming of both negative emotions and threatening events. These results supported the TST, which proposes that encountering threatening events during waking causes threatening events in dreams. Moreover, dreams with threatening events were more emotional and negative than dreams without threatening events. The TST can also explain these results, because it argues that the TSS can choose salient long-term autobiographical memories that increases the emotional intensity of threatening dreams. Finally, our results may give some insight into mathematical model of Schredl (2003, 2012) for the ICH.

## Data Availability Statement

The datasets used and/or analysed during the current study are available from the corresponding author on reasonable request.

## Ethics Statement

The studies involving human participants were reviewed and approved by the Committee of the South China Normal University. The patients/participants provided their written informed consent to participate in this study.

## Author Contributions

All authors listed have made a substantial, direct and intellectual contribution to the work and approved it for publication.

## Conflict of Interest

The authors declare that the research was conducted in the absence of any commercial or financial relationships that could be construed as a potential conflict of interest.

## Publisher’s Note

All claims expressed in this article are solely those of the authors and do not necessarily represent those of their affiliated organizations, or those of the publisher, the editors and the reviewers. Any product that may be evaluated in this article, or claim that may be made by its manufacturer, is not guaranteed or endorsed by the publisher.
